# Editorial: New Strategies to Combat Antimicrobial Resistance in Infectious Diseases

**DOI:** 10.3390/ph19020274

**Published:** 2026-02-06

**Authors:** Antonia Efstathiou, Dimitra K. Toubanaki

**Affiliations:** Immunology of Infection Group, Department of Microbiology, Hellenic Pasteur Institute, 11521 Athens, Greece; dtouban@pasteur.gr

One of the greatest challenges of our era, and of modern medicine, is the accelerating rise of antimicrobial resistance (AMR). This outcome is mainly a result of infections which were once successfully treated but which are now becoming increasingly unmanageable, therapeutic options which are narrowing, and the innovation pipeline which remains lagging. In 2023, the World Health Organization (WHO) published a global research agenda comprising 40 priority topics for AMR, where the need for innovation in diagnostics, therapeutics, diseases management, and the One Health paradigm are highlighted [1]. However, up until 2025, pivotal gaps remain in surveillance and national actions for plans’ implementation [2]. Alternative strategies, namely vaccines, antimicrobial peptides (AMPs), nanotechnology-based delivery systems, and system-level approaches, are moving toward translation and promise to help fight this great threat. Specifically, vaccines targeting main bacterial pathogens offer a high-impact route to reduce antibiotic use and thereby slow the emergence of resistance [3,4]. The research collected in this Special Issue of *Pharmaceuticals* brings together original research articles, reviews, and systematic analyses addressing novel antimicrobials, resistance mechanisms, combination therapies, in silico omics approaches, and translational strategies and thus provides an important snapshot of where the field stands and where it must go.

A representative selection of contributions in this Special Issue illustrates the breadth and scientific depth of contemporary ways to combat antimicrobial resistance. For instance, K.S. Allemailem presents a robust omics-driven and immunoinformatics-based approach for the development of vaccine against *Helcococcus kunzii*, an emerging opportunistic pathogen with increasing clinical relevance and antibiotic resistance. In this article, the integration of subtractive proteomics, reverse vaccinology, and immune simulation, demonstrates how computational pipelines can accelerate antigen discovery and rational vaccine design, and reduce experimental costs while improve translational feasibility. Overall, this study highlights the growing importance of in silico methodologies in protective strategies to fight against resistant pathogens [5].

In the context of novel antimicrobial discovery, Balaes et al. present the synthesis and biological evaluation of hybrid bis-(imidazole/benzimidazole)-pyridine derivatives with significant antifungal activity against both human and phytopathogenic fungi [6]. This study has a strong methodological innovation, which integrates ultrasound-assisted synthesis to enhance efficiency, while maintaining a strong emphasis on sustainability and comprehensive antimicrobial profiling. Importantly, the demonstrated activity against diverse fungal strains underscores the translational relevance of these compounds for both medical and agricultural applications, addressing antifungal resistance from a One Health perspective.

Strukova et al. address one of the most urgent clinical AMR threats by investigating the potent antibacterial action of the meropenem–avibactam combination therapy against carbapenemase-producing *Klebsiella pneumoniae* [7]. The pharmacokinetically informed experimental design, which employs an in vitro hollow-fibre infection model and a PK-based MIC determination approach, represents the study’s major advantage. This work confirms the therapeutic potential of β-lactam/β-lactamase inhibitor combinations and provides a methodological framework for improving antimicrobial susceptibility testing and efficacy prediction in resistant infections.

A comprehensive review by Boleti et al. presents wound healing mechanisms with a particular focus on antimicrobial peptides (AMPs) and emerging regenerative technologies [8]. This review effectively integrates molecular and cellular mechanisms with applied innovations, namely smart biomaterials, nanotechnology, and gene therapy. Its strength lies in positioning AMPs as antimicrobial agents as well as immunomodulatory and pro-regenerative molecules and thus highlight their therapeutic relevance in chronic and biofilm-associated infections.

Further focusing on innovative anti-AMR strategies, Jacobowski et al. deliver a pioneering review that combines advances in engineered peptides, nanotechnology-based drug delivery, bacteriophage therapy, quorum-sensing interference, and CRISPR-Cas systems [9]. This work stands out for its system-level perspective, critically evaluating the translational challenges but also the perspectives of next-generation antimicrobials. The authors emphasize resistance-proof strategies and targeted interventions, thus establishing this review as a valuable conceptual roadmap for future antimicrobial development.

Finally, the systematic review by Zouganeli et al. offers a population-level and epidemiological dimension to the Special Issue by examining the impact of the COVID-19 pandemic on drug-resistant tuberculosis in Europe [10]. Drawing on multi-year data from the European Centre for Disease Prevention and Control, this meta-analysis highlights how healthcare disruptions can amplify resistance trends, particularly for MDR and rifampicin-resistant tuberculosis. The strength of this study lies in its public health relevance, reinforcing the essential role of surveillance, stewardship, and sustained investment in diagnostic and treatment infrastructures to reduce long-term AMR consequences.

Overall, three overarching themes emerge from the contributions published in this Special Issue, collectively framing current progress and future directions in the fight against antimicrobial resistance:

(i) Targeting the resistant microorganism via non-classical mechanisms

Conventional antimicrobial strategies have historically focused on inhibiting essential cellular mechanisms such as cell wall biosynthesis, protein translation, or nucleic acid replication. However, widespread resistance mechanisms, including β-lactamase production, reduced membrane permeability, target modification, and biofilm formation, have substantially diminished the efficacy of many frontline therapies. Several studies in this Special Issue highlight alternative approaches that move beyond these classical paradigms. In particular, the work by Moldovan et al. demonstrates that newly synthesized tricyclic flavonoids exhibit potent activity against multidrug-resistant *Staphylococcus aureus*, with experimental evidence supporting bacterial membrane impairment as a key mechanism of action [11]. By targeting membrane integrity rather than canonical intracellular targets, this strategy may circumvent resistance pathways associated with target site modification and reduced drug uptake. Similarly, combination therapy approaches are addressed by Strukova et al., who show that the addition of avibactam restores meropenem efficacy against carbapenemase-producing *Klebsiella pneumoniae* [7].

Collectively, these studies reflect a broader shift in AMR research toward targeting microbial adaptability and resistance phenotypes rather than relying exclusively on single-target antimicrobial inhibition, a fact that is also supported by recent external reviews.

(ii) Harnessing advanced drug-delivery and peptide-based and innovative therapeutic platforms

The development of alternative antimicrobial platforms represents a central theme of this Special Issue. Recent external literature has highlighted the growing role of nanotechnology-based approaches, including metallic nanoparticles, liposomes, dendrimers, and nanozymes, in combating multidrug-resistant pathogens through membrane disruption, reactive oxygen species generation, and targeted drug delivery. While original nanotechnology-based experimental studies are not the primary focus of this Special Issue, several reviews in the Special Issue provide critical conceptual and translational insights into these approaches.

Notably, Boleti et al. comprehensively review the role of antimicrobial peptides (AMPs) in wound healing and infection control, emphasizing their dual antimicrobial and immunomodulatory functions [8]. The review highlights how AMPs can disrupt biofilms, modulate host immune responses, and promote tissue regeneration, positioning them as promising candidates for chronic and hard-to-treat infections. Complementing this perspective, Jacobowski et al. discuss a broad spectrum of innovative anti-AMR strategies, including peptide engineering, nanotechnology-assisted drug delivery, bacteriophage therapy, quorum-sensing interference, and CRISPR-Cas-based approaches [9]. A key strength of this review lies in its balanced evaluation of both therapeutic potential and translational challenges, such as toxicity, stability, manufacturing scalability, and regulatory hurdles.

Together, these contributions underscore the importance of platform-based and mechanism-diverse strategies in expanding the antimicrobial armamentarium beyond traditional antibiotics.

(iii) Prevention and system-level approaches: vaccines, omics, and One Health perspectives

Prevention remains one of the most effective and sustainable strategies for reducing antimicrobial resistance. In particular, vaccination plays a critical role in decreasing infection incidence, limiting antibiotic use, and interrupting transmission of resistant pathogens. Recent analyses by the World Health Organization have estimated that optimized vaccine deployment could substantially reduce global antibiotic consumption. Within this Special Issue, preventive strategies are addressed through both molecular and population-level perspectives.

Allemailem presents an omics-driven, immunoinformatics-based framework for vaccine target discovery against *Helcococcus kunzii*, an emerging opportunistic pathogen with increasing resistance to conventional antibiotics. By integrating subtractive proteomics and reverse vaccinology, this study illustrates how computational approaches can accelerate antigen identification and rational vaccine design, supporting precision prevention strategies [5].

At the population level, Zouganeli et al. provide a systematic analysis of the impact of the COVID-19 pandemic on drug-resistant tuberculosis in Europe, as presented above [10]. Their findings highlight how disruptions in healthcare delivery, diagnostics, and treatment continuity can exacerbate resistance trends, reinforcing the importance of resilient surveillance systems and antimicrobial stewardship. In parallel, several reviews and original studies in this Special Issue address resistance across bacterial, fungal, and parasitic pathogens, reinforcing a One Health perspective in which human, animal, and environmental reservoirs of resistance are intrinsically interconnected.

While the contributions in this Special Issue advance the field substantially, several critical gaps remain and warrant coordinated future efforts:

- Translation to clinical practice: Promising laboratory-based innovations, including antimicrobial peptides, combination therapies, and advanced delivery platforms, must be accompanied by rigorous toxicity assessment, scalability, cost-effectiveness, and clinical validation to enable real-world implementation.
- Resistance evolution to novel modalities: Even non-classical approaches such as membrane-targeting agents, peptides, or nanomaterials may exert selective pressure. Continuous surveillance and evolutionary modelling are essential to anticipate and reduce resistance emergence.
- Expanded deployment of vaccines and diagnostics: Despite growing evidence supporting their role in AMR mitigation, vaccines and rapid diagnostics remain underutilized in many regions, particularly in low- and middle-income countries, underscoring the need for equitable access and policy integration.
- Strengthened One Health surveillance: Resistance surveillance across environmental, agricultural, and human health sectors remains fragmented. Integrated data-sharing frameworks are urgently needed to capture transmission dynamics at the human–animal–environment interface.
- Integration of omics and systems pharmacology: Multi-omics approaches offer powerful tools for target discovery, resistance monitoring, and therapeutic optimization, yet their routine integration into antimicrobial research and clinical workflows remains limited. Bridging this gap will be essential for developing durable, precision-guided anti-infective strategies.

In conclusion, this Special Issue of *Pharmaceuticals* offers a curated and timely collection of work at the intersection of antimicrobial discovery, resistance mitigation, drug-delivery innovation, and preventive strategies. Together, the contributions echo a unified message: combating AMR demands multifaceted solutions that transcend conventional antibiotic discovery and stewardship. As guest editors, we extend our sincere thanks to all authors, reviewers, and the editorial team for their efforts in bringing this issue to fruition. It is our hope that the insights presented herein will stimulate further research, catalyze translational breakthroughs, and strengthen the global response to AMR.

We look forward to a future where vaccines, new molecules, smart delivery systems, and integrated surveillance form a coherent arsenal against the threat of drug-resistant infections. The path is challenging but increasingly defined. Let this Special Issue mark a step forward in that journey.

## References

[1] WHO (2023). WHO Outlines 40 Research Priorities on Antimicrobial Resistance. https://www.who.int/news/item/22-06-2023-who-outlines-40-research-priorities-on-antimicrobial-resistance.

[2] WHO (2024). Better Use of Vaccines Could Reduce Antibiotic Use by 2.5 Billion Defined Daily Doses Annually. https://www.who.int/news/item/10-10-2024-better-use-of-vaccines-could-reduce-antibiotic-use-by-2.5-billion-doses-annually--says-who.

[3] Mouzakis A., Panagopoulos P., Papazoglou D., Petrakis V. (2025). A Comprehensive Review of Nanoparticles in the Fight Against Antimicrobial Resistance. Pathogens.

[4] Desai A., Ghosh S., Sankaranarayanan S., Bhatia D., Yadav A.K. (2025). A one health nanotechnology approach to address antimicrobial resistance: State-of-the-art and strategic outlook. Mater. Adv..

[5] Allemailem K.S. (2025). In Silico Development of a Chimeric Multi-Epitope Vaccine Targeting *Helcococcus kunzii*: Coupling Subtractive Proteomics and Reverse Vaccinology for Vaccine Target Discovery. Pharmaceuticals.

[6] Balaes T., Mangalagiu V., Antoci V., Amariucai-Mantu D., Diaconu D., Mangalagiu I.I. (2025). Hybrid *Bis*-(Imidazole/Benzimidazole)-Pyridine Derivatives with Antifungal Activity of Potential Interest in Medicine and Agriculture via Improved Efficiency Methods. Pharmaceuticals.

[7] Strukova E.N., Portnoy Y.A., Golikova M.V., Zinner S.H. (2024). In Vitro Dynamic Model Evaluation of Meropenem Alone and in Combination with Avibactam Against Carbapenemase-Producing *Klebsiella pneumoniae*. Pharmaceuticals.

[8] Boleti A.P.d.A., Jacobowski A.C., Frihling B.E.F., Cruz M.V., Santos K.F.D.P., Migliolo L., de Andrade L.R.M., Macedo M.L.R. (2025). Wound Healing: Molecular Mechanisms, Antimicrobial Peptides, and Emerging Technologies in Regenerative Medicine. Pharmaceuticals.

[9] Jacobowski A.C., Boleti A.P.A., Cruz M.V., Santos K.F.D.P., de Andrade L.R.M., Frihling B.E.F., Migliolo L., Paiva P.M.G., Teodoro P.E., Teodoro L.P.R. (2025). Combating Antimicrobial Resistance: Innovative Strategies Using Peptides, Nanotechnology, Phages, *Quorum Sensing* Interference, and CRISPR-Cas Systems. Pharmaceuticals.

[10] Zouganeli C., Toubanaki D.K., Karaoulani O., Vrioni G., Karagouni E., Efstathiou A. (2025). Impact of the COVID-19 Pandemic on Drug-Resistant Tuberculosis in Europe: A Meta-Analysis of Epidemiological Trends. Pharmaceuticals.

[11] Moldovan C.-V., Mantea L.-E., Savu M., Jones P.G., Sarbu L.G., Stefan M., Birsa M.L. (2024). Novel Tricyclic Flavonoids as Promising Anti-MRSA Agents. Pharmaceuticals.

